# Micro‐CT assessment of dental mineralization defects indicative of vitamin D deficiency in two 17th–19th century Dutch communities

**DOI:** 10.1002/ajpa.23819

**Published:** 2019-03-18

**Authors:** Barbara Veselka, Megan B. Brickley, Lori D'Ortenzio, Bonnie Kahlon, Menno L. P. Hoogland, Andrea L. Waters‐Rist

**Affiliations:** ^1^ Faculty of Archaeology, Human Osteoarchaeology Laboratory Leiden University Leiden The Netherlands; ^2^ Department of Anthropology McMaster University Hamilton Ontario Canada; ^3^ Department of Anthropology Western University London Ontario Canada

**Keywords:** interglobular dentine, residual rickets, rickets, seasonality, Western Europe

## Abstract

**Objectives:**

This study investigates vitamin D deficiency patterns in individuals from birth to the beginning of adolescence. Microscopic computed tomography (micro‐CT) evaluation of interglobular dentine (IGD) in teeth provides information on the age of disease onset and the number of deficient periods per individual, which will increase our understanding of factors influencing vitamin D deficiency prevalence, including sociocultural practices and latitude.

**Materials and methods:**

Beemster and Hattem, two Dutch 17th–19th century communities, yielded relatively high prevalences of rickets (15–24%) and residual rickets (15–24%). From the affected individuals, a subsample of 20 teeth were selected for micro‐CT scanning. Thin sections were made of 17 teeth, consisting of 6 teeth with and 11 teeth without observable IGD on micro‐CT that were included for method comparison.

**Results:**

About 19 out of 29 (65.5%) individuals (one tooth was deemed unobservable) presented with IGD on micro‐CT. Eight of the 11 (72.7%) individuals without IGD on micro‐CT demonstrated histologically visible IGD. In 40.7% (11/27) of the affected individuals (combined micro‐CT and histology results), vitamin D deficiency was recurrent, and in four individuals, some episodes occurred at approximately annual intervals suggesting vitamin D deficiency was seasonal. In three individuals, IGD occurred in the dentine formed around birth, suggesting maternal vitamin D deficiency.

**Discussion:**

Micro‐CT analysis of IGD is found to be a valuable non‐destructive method that can improve our understanding of the influence of sociocultural practices and latitude on disease development within age and sex groups in past communities.

## 1 INTRODUCTION

Vitamin D is important in the evolution of human skin pigmentation, growth, and health (Eckhardt, Gernand, Roth, & Bodnar, 2015; Holick, 2004; Jablonski & Chaplin, 2013, 2018). Vitamin D plays an important role in the calcium homeostasis needed for mineralization of osteoid, newly formed bone tissue (Holick, 2006). Mineralization ensures structural integrity of the skeleton enabling it to withstand gravity and muscular tension. Cutaneous production of vitamin D under the influence of ultraviolet B (UVB) radiation in sunlight is the most effective way of obtaining an adequate amount of vitamin D, with foods such as oily fish providing supplemental amounts (Brickley, Moffat, & Watamaniuk, 2014; Holick, 2003, 2006). Insufficient sunlight exposure will lead to vitamin D deficiency and bending deformities of the skeleton may develop that are visible in archeological human remains (Brickley et al., 2014; Brickley, Mays, George, & Prowse, 2018; Brickley, Mays, & Ives, 2010; Mays, Brickley, & Ives, 2006; Ortner & Mays, 1998). Improved methods for detecting vitamin D deficiency, and reconstructing the number of deficiency events and their age of occurrence, will open up multiple avenues to better understand the relationship between vitamin D and human adaptation and evolution.

Recent research has shown that skeletal evidence of vitamin D deficiency provides important information on sociocultural practices related to sunlight exposure and diet, and can aid in the reconstruction of past lifeways (Brickley et al., 2014; Giuffra et al., 2015; Palkovich, 2012; Veselka, Hoogland, & Waters‐Rist, 2015; Waters‐Rist & Hoogland, 2018; Watts & Valme, 2018). Vitamin D deficiency in archeological remains is traditionally assessed via macroscopic examination of various lesions. This approach does not offer information about the number of separate episodes of vitamin D deficiency nor the age at which deficiency occurred. A recently developed method by D'Ortenzio et al. (2016) demonstrated the use of histological examination of dental mineralization defects called interglobular dentine (IGD) as a marker of vitamin D deficiency. A recent clinical investigation has shown that IGD is visible on microscopic computed tomography (micro‐CT) scans (Ribeiro, Costa, Soares, Williams, & Fonteles, 2015) and Colombo et al. (2019) have shown IGD can also be observed in archeological teeth. IGD analysis via non‐destructive micro‐CT can enable the determination of the age of vitamin D deficiency onset and the frequency of vitamin D deficient periods within an individual (Colombo et al., 2019).

This study applies micro‐CT analysis of IGD to aid in the diagnosis of vitamin D deficiency. This article will use micro‐CT IGD analysis to investigate parameters of vitamin D deficiency in a subset (*n* = 30) of two previously studied 17th–19th century Dutch communities, Beemster (MB11) and Hattem (HT15) that were shown to have high levels of rickets (between 15% and 24%) and residual rickets (between 14% and 24%) (Veselka, 2019; Veselka et al., 2015; Veselka, Hoogland, & Waters‐Rist, 2018). Progress has been made in paleopathology vitamin D deficiency research, for example in research that has begun to differentiate between cases of healing and active rickets (Brickley et al., 2018; Mays et al., 2006), however, the development of vitamin D deficiency in various age groups is still poorly understood, and the influence of sex and gender roles in nonadults is rarely investigated (Veselka et al., 2015). IGD research offers an opportunity to remedy these gaps in our knowledge.

The objectives of this article are, firstly, to investigate vitamin D deficiency patterns in individuals from birth to the beginning of adolescence via the assessment of the age of disease onset and the number of deficient periods per individual in two post‐Medieval Dutch communities. Information on the age of disease onset improves our understanding of vitamin D deficiency development and may provide information on sociocultural practices that possibly increased the risk of deficiency or that exacerbated the condition. The number of IGD formation periods provides information on individual differences in disease duration and consecutive bands of IGD may be indicative of seasonality. Our second aim is to investigate the potential of micro‐CT analysis of vitamin D deficiency by comparing macroscopic, micro‐CT, and histological assessment of this condition. This study is the first to apply a wide‐scale assessment of macroscopic, micro‐CT, and histological methods of vitamin D deficiency diagnosis.

## 2 MATERIALS AND METHODS

**Figure 1:**
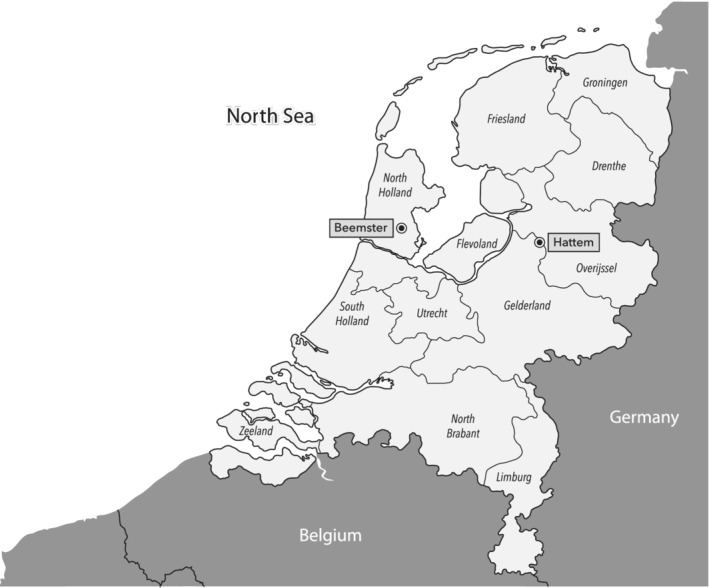
Location of Beemster and Hattem in the Netherlands

Two communities were assessed for this article (a) Beemster (MB11), a rural settlement dating to the 19th century in the province of North‐Holland with a skeletal sample consisting of 59 nonadults and 200 adults, and (b) Hattem (HT15), a small urban center dating to the 17th–19th centuries in the province of Gelderland with a skeletal sample consisting of 21 nonadults and 88 adults. Figure 1 shows the location of these sites.

Macroscopic analysis of both skeletal collections was undertaken prior to this study (Veselka, 2019; Veselka et al., 2015, 2018). Rickets, active and healing vitamin D deficiency in nonadults (<18 years of age), was assessed by scoring various skeletal lesions, including flaring and/or cupping of the long‐bone metaphyses, porosity underlying the growth plate, and bending deformities of the long bones (Brickley & Ives, 2008; Mays et al., 2006; Ortner & Mays, 1998). Residual rickets was scored as remnant lesions of a nonadult vitamin D deficiency in the adult skeleton, using the criteria described by Brickley and Ives (2008) and Brickley et al. (2010). Rickets prevalence in the Beemster community was 15.3% (9/59) and 23.8% (5/21) in Hattem. Residual rickets prevalence was 14.5% (29/200) in Beemster and 23.9% (21/88) in Hattem (Veselka, 2019; Veselka et al., 2015, 2018). A subset of 30 affected individuals (out of 64) was selected for micro‐CT IGD analysis, 15 from each site, including nonadults and adults, and both sexes. The selected individuals with their basic demographic information are presented in Table 1 in the Results section. For each site we attempted to select a roughly even number of males to females, and nonadults to adults, however, this was not possible for the Beemster sample as none of the affected nonadults and only two males (out of the eight affected) had suitable teeth for micro‐CT scanning.

**Table 1:** Overview of analyzed individuals with their sex, age, sampled tooth, macroscopic vitamin D deficiency, number of IGD periods, and the age of IGD formation

Individual				Macro‐scopy†	Micro‐CT		Histology		
Site/ID#	Sex†	Age‐at‐death (years)†	Tooth sampled	Category	N IGD periods	Age (period of deficiency)‡	N IGD periods	Grade	Age (period of deficiency)‡
HT15S020	U	15 ± 2	RM_1_	Possible	0	–	1	1	6–12 mths.
HT15S042	M	36–49	LM^1^	Present	1	2.5 yrs.	1	2	2.5 yrs.
HT15S062	F	36–49	LM_1_	Present	0	–	0	–	–
HT15S066	M	36–49	LM_1_	Present	1	6–12 mths.	NA	–	–
HT15S067	U	6.5 ± 0.5	LM^1^	Present	2	6–12 mths.	NA	–	–
						2.5 yrs.			
HT15S071	M	18–25	RM_1_	Present	0	–	0	–	–
HT15S075	F	36–49	LC^1^	Present	U	U	3	2 and 3	2.5 yrs. 5 yrs. 6 yrs.
HT15S080	M	26–35	RM^1^	Present	2	6–12 mths.	NA	–	–
						2.5 yrs.			
HT15S094	F	18–25	RM_1_	Present	2	6–12 mths.	2	2	6–12 mths. 2.5 yrs.
						3 yrs.			
HT15S099	F	18–25	LM^1^	Present	0	–	1	1–2	6–12 mths.
HT15S106	M	36–49	RM_1_	Present	1	6–12 mths.	NA	–	–
HT15S109	F	36–49	RM^1^	Present	0	–	1	1–2	2.5 yrs.
HT15S123	U	2 ± 0.5	RM^1^	Present	1	6–12 mths.	NA	–	–
HT15S127	U	9 ± 1	RM_1_	Present	2	Birth	NA	–	–
						6–12 mths.			
HT15S130	M	18–25	RC^1^	Present	4	12 mths.	6	2 and 3	1.5 yrs. 2 yrs. 2.5 yrs. 3.5 yrs. 6.5 yrs. 7.5 yrs.
						2.5 yrs.			
						3 yrs.			
						5 yrs.			
MB11S101	F	26–35	RM^1^	Present	2	6–12 mths.	3	2 and 3	6–12 mths. 2 yrs. 3 yrs.
						2.5 yrs.			
MB11S126	F	36–49	RM^1^	Possible	0	–	1	1–2	Birth – 12 mths.
MB11S183	F	26–35	LM^1^	Present	0	–	1	1–2	6–12 mths.
MB11S234	F	18–25	RM^1^	Present	0	–	1	2–3	6–12 mths.
MB11S307	F	18–25	RM^1^	Present	4	Birth	4	2 and 3	1 yr. 2 yrs. 3 yrs. 5 yrs.
						6–12 mths.			
						2.5 yrs.			
						5 yrs.			
MB11S321	M	50+	RM^1^	Present	0	–	0	–	–
MB11S327	F	26–35	RM^1^	Present	2	6–12 mths.	NA	–	–
						2.5 yrs.			
MB11S401	F	26–35	LC_1_	Present	2	2.5 yrs.	NA	–	–
						3 yrs.			
MB11S413	F	36–49	LM^1^	Possible	1	6–12 mths.	NA	–	–
MB11S420	F	26–35	RM^1^	Present	1	2.5 yrs.	2	2–3	6–12 mths. 2.5 yrs.
MB11S422	F	36–49	RM^1^	Present	1	Birth – 12 mths.	NA	–	–
MB11S427	M	26–35	RM^1^	Present	1	6–12 mths.	NA	–	–
MB11S437	F	26–35	LM^1^	Present	2	6–12 mths.	NA	–	–
						2.5 yrs.			
MB11S488	F	36–49	RM_1_	Present	1	2.5 yrs.	NA	–	–
MB11S498	F	50+	LC^1^	Present	0	–	1	1–2	2.5 yrs.

† Data from Veselka et al. (2015, 2018) and Veselka (2019).

‡ Age is based on Moorrees, Fanning, and Hunt (1963) and Massler, Schour, and Poncher (1941), age periods are approximate. U = unobservable, M = male, F = female, RM^1^ = right first permanent maxillary molar, LM^1^ = left first permanent maxillary molar, RM_1_ = right first permanent mandibular molar, LM_1_ = left first permanent mandibular molar, RC^1^ = right permanent maxillary canine, LC^1^ = left permanent maxillary canine, LC_1_ = left permanent mandibular canine, mths. = months, yrs. = years (all ages approximate), HT15 = Hattem, MB11 = Beemster, IGD = interglobular dentine, NA = not assessed.

The first permanent molar was selected for micro‐CT assessment if available and if dental wear did not exceed stage H (Lovejoy, 1985), indicating the dentine underlying the enamel was still intact. If the first permanent molar was not available (worn or absent), the permanent canine was sampled if available and dental wear did not exceed stage D (Lovejoy, 1985), ensuring the dentine would be observable. In the first permanent molar, dentine formation starts in utero and will enable IGD assessment up to 10 years of age, while in the permanent canine, dentine formation starts around 3 months of age and ends at about 14 years (Gustafson & Koch, 1974; Massler et al., 1941; Moorrees et al., 1963).

Dentine is formed is two phases, whereby odontoblasts secrete predentine which is then mineralized (Beaumont, Gledhill, Lee Thorp, & Montgomery, 2013; Bevelander & Nakahara, 1966; Hillson, 2002). If there are adequate nutritional conditions during the processes of formation and calcification, the matrix will appear homogenous and fusion of calcospherites, spheres containing calcium salts, will be complete (D'Ortenzio et al., 2016; Hillson, 2002). However, when vitamin D levels are inadequate, some of the calcospherites fail to fuse, which is visible as poorly mineralized patches of dentine with a bubble‐like appearance, referred to as IGD (D'Ortenzio et al., 2016; Isokawa, Kosakai, & Kajiyama, 1963; Vital et al., 2012). The presence of IGD is clinically associated with conditions disrupting mineralization by affecting vitamin D, calcium and/or phosphate levels (Chaussain‐Miller et al., 2003; McDonnell, Derkson, Zhang, & Hlady, 1997; Souza, Soares, Alves dos Santos, & Vaisbisch, 2010; Vital et al., 2012).

Based on histological data, D'Ortenzio et al. (2016) divide IGD into three grades of severity from 1 to 3. However, grading of IGD severity based on micro‐CT images was not undertaken in this study because recent research has suggested features of IGD are less clear on micro‐CT (Colombo et al., 2019). Micro‐CT detects differences in mineralization density and since the bubble‐like spaces in grade 1 are relatively small, is it suggested that grade 1 is not visible on micro‐CT scan (Colombo et al., 2019). Instead, in this study, IGD on micro‐CT is scored as “present”, “absent”, or “unobservable”. If present, IGD will be visible as bands of dispersed micro‐defects that can be distinguished from taphonomic degradation, because bands of IGD will follow the incremental lines found in dentine and isolated micro‐defects are more likely to be taphonomic or developmental defects. If patches of degradation or cracks are present and mimic bands, these are easily distinguished from IGD by switching to other views, for instance craniocaudal versus transverse.

In this study, the age at which an episode of IGD occurred is estimated using the criteria of Massler et al. (1941) and Moorrees et al. (1963). Dentine grows in concentric cones (Eerkens, Berget, & Bartelink, 2011; Hillson, 2002) at rate of about 4–6 μm a day in permanent teeth. The first mm of the dentine below the crown in the first permanent molar represents the period around birth to about 1.5 years of age while the root dentine begins forming around 4 years of age (Beaumont et al., 2013; Gustafson & Koch, 1974; Massler et al., 1941; Moorrees et al., 1963).

Micro‐CT scans were performed using a SkyScan 1272 (100 kV, 100 μA, Cu 0.11 mm, 10.0 μm). The scan was reconstructed using NRecon^©^ software. The 3D reconstruction was assessed and when IGD was present, a section image from each plane (buccal–lingual, mesial–distal, and transverse) was made. The occurrence and number of IGD bands were assessed by all researchers independently and only defects which were classified by all researchers as having IGD were included.

Since IGD grade 1 is not expected to be detectable on micro‐CT, a total of 17 thin sections for histological assessment were made from all 10 individuals that did not display IGD on micro‐CT, for one individual where observation for IGD was not possible (discussed below), and for six individuals that did have IGD on micro‐CT for comparative purposes. The teeth were embedded in resin (EpoThin^©^) after which a thin section was made with an IsoMet® 1000 precision saw. After further grinding and polishing as described by De Boer, Aarents, and Maat (2013), the thin section was mounted on a glass slide for histological analysis using a Leica® DM500 compound microscope with 40× magnification. The number and the severity of IGD periods were scored for each tooth, whereby grading of IGD was evaluated according to D'Ortenzio et al. (2016).

A chi‐square test was performed to test the difference in the number of IGD periods per individual, the difference in the age of occurrence of the first episode of IGD, and to assess the statistical significance of the difference in the occurrence of IGD bands in specific age periods per individual. A Mann–Whitney *U‐*test was performed to assess the statistical significance of the number of IGD episodes per site and the difference in the number of IGD episodes between males and females. Results were considered statistically significant if *p* < .05.

## 3 RESULTS

Table 1 provides an overview of all individuals whose teeth were selected for micro‐CT scanning. For each individual basic demographic data is noted along with macroscopic vitamin D deficiency diagnosis, the number of IGD episodes, and the approximate age of formation of each IGD episode from both micro‐CT and histological analyses. Grading of IGD is noted for the thin sections. Appendix A provides details of the macroscopic lesions attributed to vitamin D deficiency for each individual.

**Figure 2:**
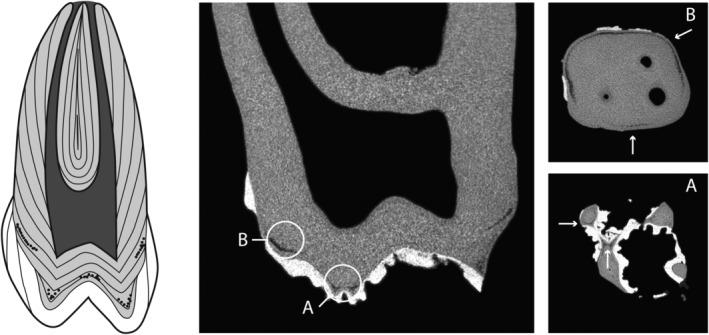
HT15S067 shows 2 episodes of IGD (a and b), visible in the schematic drawing, the micro‐CT scan in craniocaudal view, and both episodes in transverse view marked by white arrows

Nineteen individuals displayed bands of IGD on micro‐CT, with the majority (89.5%; 17/19) displaying one or two episodes. Figure 2 displays a right first permanent maxillary molar (RM^1^) of individual HT15S067 showing the location of two episodes of IGD.

As mentioned, most individuals with observable IGD on micro‐CT display one or two episodes, whereby the difference in the number of IGD bands per individual is statistically significant (*χ* ^2^ = 13.586, *p* = .009). No statistically significant difference was observed in the number of vitamin D deficient periods per individual when comparing the two sites (Hattem: mean number of IGD periods = 1.20; Beemster: mean number of IGD periods = 1.21; *U* = 104.500, *p* = .983). More than half of the affected individuals (63.2%; 12/19) displayed their first observable episode of vitamin D deficiency between 6 and 12 months of age (*χ* ^2^ = 8.103, *p* = .044). Three individuals (15.7%; 3/19) had IGD in the earliest layers that formed around birth. IGD was most frequently observed (*χ* ^2^ = 10.862, *p* = .012) in the age period of 6–12 months (78.9%; 15/19). The majority of individuals (89.5%; 17/19) did not display IGD bands after about 2.5–3 years of age.

**Figure 3:**
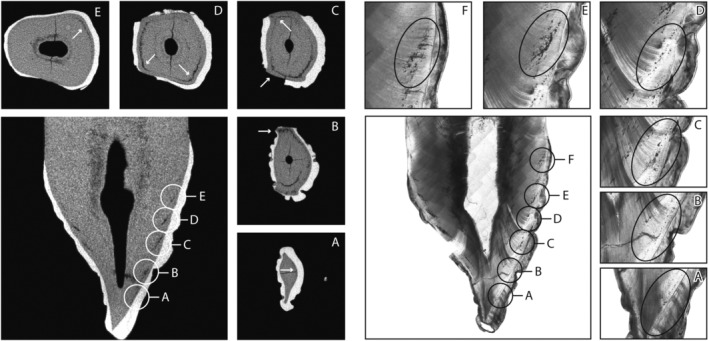
Craniocaudal micro‐CT image of the right permanent maxillary canine (RC^1^) of individual HT15S130 with corresponding transverse image of each IGD episode marked by white arrows, and a thin section image at 40x magnification of the same tooth with each of the IGD episodes shown separately

About 11 out of 17 individuals (64.7%; 11/17) displayed more IGD episodes on thin section than on micro‐CT (including HT15S075 which was considered unobservable on micro‐CT), whereas the remaining six individuals displayed the same number of IGD bands on micro‐CT and on thin section. Figure 3 shows a craniocaudal micro‐CT image of the right permanent maxillary canine (RC^1^) of individual HT15S130 demonstrating 4, and possibly 5, episodes of IGD (A–E). It was difficult to determine on micro‐CT if episodes A and B occurred separately or together over a longer continuous period. Therefore, the fifth episode of IGD was considered to be possibly present. On the histological image of the same tooth, the same episodes are visible (A–E) and one additional period (F) that is not visible on micro‐CT. In episode A of the histological image, two bands of IGD are clearly visible within the same layer of dentin suggesting to be part of the same period, which could be the result of differences in appositional growth of the dentin throughout the layer (D'Ortenzio, Kahlon, Peacock, Salahuddin & Brickley, 2018).

**Figure 4:**
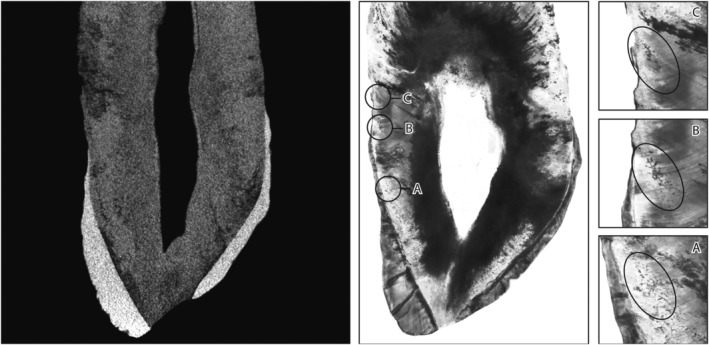
Craniocaudal image of left maxillary canine of HT15S075 via micro‐CT (LC^1^). It was not possible to distinguish cracks and diagenetic degradation from bands of IGD. The histological image of the same tooth permits the observation of three IGD bands, marked as a, c, and c at 40× magnification

Figure 4 displays the left maxillary canine of HT15S075 on micro‐CT. Due to extensive degradation of the tooth, it is not possible to distinguish diagenetic damage from bands of IGD with micro‐CT, whereas on thin section, three bands of IGD were observed.

Three out of the 30 individuals (10%) that displayed macroscopic lesions that were (possibly) attributed to vitamin D deficiency, did not present with IGD episodes after micro‐CT or histological assessment. As described in the Appendix, these individuals (HT15S062, HT15S071, and MB11S321) have bending deformities of both tibiae. Individual HT15S062 also displayed bowing of the left femur, and HT15S071 presented with coxa vara of both femora.

Three different individuals were previously macroscopically diagnosed as having possible residual rickets due to only partially meeting the criteria described by Veselka et al. (2018). HT15S020 displayed anterolateral bending of both femora, MB11S126 showed bending deformities of both radii and ulnae, and MB11S413 presented with bending deformities of the left femur and the left radius. All three presented with episodes of IGD; dentine defects were visible after micro‐CT in MB11S413 and histological analysis in HT15S020 and MB11S126.

The difference in IGD prevalence on micro‐CT between Hattem females (1/4) and males (5/6) is not statistically significant (Fisher's exact test: *p* = .190), which may be partially attributed to small sample size. It is worth noting that the majority of Hattem females (75.0%; 3/4) did not display visible bands of IGD on micro‐CT, whereas the majority of the males displayed one or more IGD episodes (83.3%; 5/6). It was not possible to assess differences between the prevalence of IGD in Beemster females and males, because only two males had observable teeth.

## 4 DISCUSSION

### 4.1 Macroscopy, micro‐CT, and histology

Dentine and bone are mineralized tissues that display similarities in their formation and composition, and will react similarly to pathological conditions affecting mineralization (D'Ortenzio et al., 2016; Foster, Nociti, & Somerman, 2014; Vital et al., 2012). Vitamin D plays a vital role in the mineralization process of both teeth and bone, and a deficiency will disrupt this process. Vitamin D deficiency may lead to bending deformities of the long bones, changes to the metaphyses (flaring, cupping, and/or thickening) and sternal rib ends (thickening, and/or increased porosity), and other skeletal deformities in the growing skeleton (Brickley et al., 2014, 2018; Mays et al., 2006; Ortner & Mays, 1998). However, bone is a dynamic tissue that undergoes continuous remodeling, and if vitamin D deficiency is overcome, less severe bending deformities and skeletal lesions will remodel and may be completely obliterated (Brickley et al., 2017; Hess, 1930). Indeed, of those that do develop visible bending deformities of the long bones during childhood, only 10–15% are estimated to remain visible in the adult skeleton due to growth and the process of remodeling (Brickley et al., 2017; Hess, 1970). In contrast, dentine does not turnover and poorly mineralized dentine will remain visible even after a vitamin D deficiency is overcome (D'Ortenzio et al., 2016; Foster et al., 2014; Vital et al., 2012). Moreover, micro‐CT assessment of IGD is useful because not all individuals that experience an episode of vitamin D deficiency will develop lesions that can be identified via macroscopic examination (D'Ortenzio, Ribot, et al., 2018). Micro‐CT assessment of IGD can also be applied to individuals with missing skeletal elements. Poor skeletal preservation and incompleteness of the skeleton have been shown to affect diagnosis in nonadults, and assessment of past vitamin D deficiency in adults may be hindered when long bones and ribs are unobservable since bending deformities of these skeletal elements are the most frequently observed (Brickley et al., 2018).

More than half of the individuals with macroscopic lesions due to rickets and residual rickets, with teeth available for scanning, presented with one or more vitamin D deficient periods visible as episodes of IGD on micro‐CT (65.5%; 19/29). Histological assessment of 17 individuals demonstrated additional bands of IGD in 64.7% (11/17) of them. The combined results of micro‐CT and histological analysis demonstrated that 90.0% (27/30) of the individuals with macroscopic lesions attributed to past episodes of vitamin D deficiency, displayed one or more episodes of IGD. This suggests that macroscopic assessment of vitamin D deficiency is relatively reliable. However, three individuals with macroscopic lesions attributed to vitamin D deficiency, did not display IGD on micro‐CT or thin section. Bowing deformities can be produced by a wide range of pathological conditions (Brickley & Ives, 2008, Table 5.8) and as discussed by Brickley and co‐workers (2018), lesion development is complex and depends on several factors including nutrition, disease, co‐occurrence, and aspects of lifeways. It is possible that these individuals did suffer from vitamin D deficiency but that the condition developed after apical closure of the root of the permanent first molars (approximately at 9–11 years of age) and canines (approximately at 12–15 years of age) (Fanning & Brown, 1971; Gustafson & Koch, 1974; Moorrees et al., 1963). It is also possible they developed vitamin D deficiency when only the tooth roots were forming and that IGD defects did not arise because the rate of appositional dentine growth in the roots of the teeth differs from the growth rate of the dentine formed near the crown which may result in the absence of IGD formation in the roots of the first permanent molar and permanent canine despite vitamin D deficiency (D'Ortenzio, Kahlon, et al., 2018). However, the bending deformities observed in these individuals may simply represent more pronounced expression of human variation, or relate to an alternative cause. While the differential diagnosis undertaken produced no clear alternate diagnosis it is always a possibility (Veselka, 2019; Veselka et al., 2018).

The use of the non‐destructive micro‐CT method to detect vitamin D deficiency can aid in the identification of the disease in individuals who lack clear lesions, as was the case with MB11S413. This individual was diagnosed with possible residual rickets due to partially meeting the criteria as described by Veselka et al. (2018). They displayed IGD on micro‐CT which supports the residual rickets diagnosis.

Extensive diagenetic change prevented assessment using micro‐CT of one individual (HT15S075), as observed in Figure 4. In this case, assessment via thin section revealed that IGD was present and relatively easy to assess, demonstrating three episodes of IGD with grade 2 and 3 that would have been visible on micro‐CT if not for the diagenetic damage.

It was reported by Colombo et al. (2019) that grade 1 IGD was not detected using micro‐CT assessment, but the current study analyzed a much larger number of individuals and results from this investigation demonstrate that depending on the micro‐CT set‐up, some individuals at the lower end of grade 2 IGD may also be missed. Most individuals without IGD on micro‐CT and the individuals that displayed additional bands of IGD on thin section, presented with IGD grade 1 and a lower grade 2 on histological examination. Thus, our results seem to support the limitation of IGD observation on micro‐CT scan to grade 2 (possibly excluding the lower end of the range) and 3 as found by Colombo et al. (2019). It should be considered that the grading of IGD severity is subjective and provides an approximate level rather than an absolute measure.

Although histological analysis of IGD appears to provide the most accurate results and the process of producing thin sections is relatively fast and inexpensive, it requires the destruction of archeological human remains that may not be desirable or possible in all skeletal collections. Furthermore, various other features not attributed to vitamin D deficiency are visible on thin sections and not on micro‐CT, such as developmental IGD (a possible result of difference in the dentine formation rate in various parts of the tooth) and marbling or dappling (variation in normal dentine; D'Ortenzio, Kahlon, et al., 2018) that may hinder pathological IGD identification. If histological examination is not possible, micro‐CT analysis of more severe IGD may aid in the identification of vitamin D deficiency in affected individuals without clear macroscopic lesions. More importantly, the 3D nature of a micro‐CT scan enables better comparison of various IGD periods than on thin section, whereby IGD can be evaluated in different planes of the tooth.

### 4.2 Number of deficient periods and age of onset

The results of micro‐CT assessment demonstrated that three individuals (10.3%; 3/29) displayed their first period of vitamin D deficiency around birth. During pregnancy, the fetus is dependent on vitamin D levels of the mother (Dror & Allen, 2010; Mulligan, Felton, Riek, & Bernal‐Mizrachi, 2010; Thandrayen & Pettifor, 2010). If the mother is vitamin D deficient, the fetus will not be able to obtain sufficient vitamin D and may present with IGD in the first layers of dentine deposited around birth. It is possible that the mothers of HT15S127, MB11S307, and MB11S422, who show IGD in the first layers of dentine, may have suffered from adult vitamin D deficiency. The prevalence of IGD in the dentine just below the enamel may be used as an indication of adult vitamin D deficiency within a population.

The majority of individuals display their first vitamin D deficient episode before the age of about 2.5 years (78.9%; 15/19) and most affected individuals (89.5%; 17/19) do not present with IGD after the age of about 3 years. However, the possibility exists that individuals experienced periods of vitamin D deficiency during formation of the dentine in the roots (D'Ortenzio, Kahlon, et al., 2018) or in adolescence which would be difficult to observe or entirely unobservable in the first permanent molar and permanent canine. Future analysis of the second and third permanent molars of the affected individuals will provide more information on periods of vitamin D deficiency in older childhood and adolescence.

Recent macroscopic analysis of Beemster and Hattem nonadults yielded active and healing cases of rickets (Veselka, 2019; Veselka et al., 2015), whereby active cases presented with underlying porosity of the growth plates (Brickley et al., 2018; Mays et al., 2006). Almost all of the active cases of rickets were observed in individuals younger than 3 years of age, whereas most healing cases (in which porosity of the growth plates was absent) were observed in older individuals. It was postulated that an increase of sunlight exposure and possibly the introduction of vitamin D‐rich foods occurred in individuals older than 3 years of age thereby decreasing the number of active cases after this age (Veselka, 2019). Although additional periods of vitamin D deficiency may be evident in the second and third molars, the results of micro‐CT analysis appear to indicate an increase in vitamin D levels after the age of about 3 years, since the majority of affected individuals (89.5%; 17/19) do not present with periods of IGD after that age.

However, this does not seem to have been the case for HT15S075, HT15S130, and MB11S307. These individuals experienced their first episode of vitamin D deficiency in the first year of their life and on thin section presented with another 2, 3, and 5 periods, respectively. The combined results of micro‐CT and histological analysis demonstrate that 40.7% (11/27) of the individuals with IGD display two or more bands of IGD indicating vitamin D deficiency to have been recurrent in both communities. Since the Netherlands has a latitude of 53°N, no dermal synthesis of vitamin D takes place in the winter months (Jablonski & Chaplin, 2013; Webb, Kline, & Holick, 1988), and vitamin D deficiency may have been somewhat seasonal, as suggested by the presence of some bands of IGD at approximately annual intervals in HT15S075 (2.5 years; 5 years; 6 years), HT15S130 (1.5 years; 2 years; 2.5 years; 3.5 years; 6.5 years; 7.5 years), MB11S101 (6–12 months; 2 years; 3 years), and MB11S307 (1 year; 2 years; 3 years; 5 years). Certainly not all episodes seem to represent consecutive years, however many do, especially in the later years. Individual changes in the diet or in sunlight exposure may have been responsible for a temporary rise in vitamin D levels visible as IGD‐free periods. Seasonal vitamin D deficiency, especially in more northern latitudes, has previously been proposed to be observable in the archeological collection from St. Martin's Birmingham from the UK (Mays, Brickley, & Ives, 2009), and the recurrent episodes of vitamin D deficiency that were observed in individuals from 15 Roman settlements across Western Europe may have been indicative of seasonality (Brickley et al., 2018). Our results provide the first clear evidence of vitamin D deficiency occurring in recurrent episodes that would fit seasonal vitamin D deficiency.

Differences in macroscopically visible rickets and residual rickets prevalence between Beemster and Hattem were not statistically significant (Veselka, 2019), and the prevalence of IGD (first episode and the number of episodes) seems to support the notion that both communities experienced a similar risk of developing vitamin D deficiency. Recent research suggested a gendered risk for developing vitamin D deficiency in the Beemster community, whereby significantly more females (21/100) than males (8/100) were affected (Veselka et al., 2018). Due to the small number of males with suitable teeth for micro‐CT assessment, comparison of IGD between Beemster females and males was not undertaken. For Hattem, no significant difference (*χ* ^2^ = 2.191, *p* = .139) between affected males and females was observed (Veselka, 2019) and the difference in IGD prevalence between Hattem males and females was not statistically significant (Fisher's exact test: *p* = .190), partially due to small sample size. It is, however, worth noting that the majority of males (83.3%; 5/6) displayed one or more IGD episode on micro‐CT, whereas the majority of females (60.0%; 3/5) did not display IGD on micro‐CT but did so on thin section. This suggests that males experienced more severe mineralization defects than females, which may be indicative of more severe periods of vitamin D deficiency in males. However, the linkages between the severity of IGD, the degree and presence of macroscopic bending deformities, and the severity of vitamin D deficiency, are complex and need further study.

## 5 CONCLUSIONS

The combined results of macroscopic, radiographic, and histological assessment of vitamin D deficiency suggested histological analysis of IGD provides the most accurate results. However, this study demonstrates that non‐destructive micro‐CT analysis of IGD is a valuable method that may aid in the identification of vitamin D deficient individuals. This is especially valuable in poorly preserved or incomplete individuals and in those with no observable, or subtle, macroscopic lesions. This method provides information on the age of onset and the number of deficient periods, allowing a more nuanced understanding of vitamin D deficiency development during growth and development. Moreover, the non‐destructive nature of this method makes it suitable to the study of precious, ancient hominine remains.

Results of micro‐CT analysis support previous findings that nonadults after the age of 3 years were likely to have experienced an increase in sunlight exposure and possibly also enjoyed a diet that contained more vitamin D. In three individuals, episodes of IGD were observed in the dentine formed around birth which suggests maternal vitamin D deficiency. Further examination of the number of IGD episodes and the attributed age periods showed vitamin D deficiency to be a recurrent condition in 40.7% (11/27) of the affected individuals and seasonal deficiency appears to have occurred in at least four individuals. This is an important finding that supports the notion that vitamin D deficiency is often seasonal in more northern latitudes.

Micro‐CT and histological analysis of IGD enables comparison of IGD severity between males and females. Although Hattem males and females are suggested to have experienced similar risks of developing vitamin D deficiency, the males display more severe IGD than females suggesting they experienced more severe periods of vitamin D deficiency. The comparison of IGD severity between males and females may provide valuable information on gendered differences. Moreover, it aids in better understanding the linkage between macroscopic lesions and IGD prevalence, and improves our knowledge of the influence of vitamin D deficiency on certain groups in past communities. Future research on the influence of the interplay of various biophysical variables (e.g., latitude, season), sociocultural factors (e.g., clothing, socioeconomic status, gendered division of labor), and aspects of diet on vitamin D deficiency prevalence using macroscopic examination, micro‐CT and histological analysis of IGD will enhance our understanding of this relationship.

## Supporting information


**Appendix S1**: Supporting information

## References

[ajpa23819-bib-0001] Beaumont, J. , Gledhill, A. , Lee Thorp, J. , & Montgomery, J. (2013). Childhood diet: A closer examination of the evidence from dental tissues using stable isotope analysis of incremental human dentine. Archaeometry, 55, 277–295. 10.1111/j.1475-4754.2012.00682.x

[ajpa23819-bib-0002] Bevelander, G. , & Nakahara, H. (1966). The formation and mineralization of dentin. Anatomical Record, 156, 303–323. 10.1002/ar.1091560306 5867109

[ajpa23819-bib-0003] Brickley, M. , D'Ortenzio, L. , Kahlon, B. , Schattmann, A. , Ribot, I. , Raguin, E. , & Betrand, B. (2017). Ancient vitamin D deficiency: Long‐term trends. Current Anthropology, 38, 420–427. 10.1086/691683

[ajpa23819-bib-0004] Brickley, M. , & Ives, R. (2008). The bioarchaeology of metabolic bone disease (Second ed., pp. 75–134). San Diego: Academic Press.

[ajpa23819-bib-0005] Brickley, M. , Mays, S. , George, M. , & Prowse, T. L. (2018). Analysis of patterning in the occurrence of skeletal lesions used as indicators of vitamin D deficiency in subadult and adult skeletal remains. International Journal of Paleopathology, 23, 43–53. 10.1016/j.ijpp.2018.01.001 30573165

[ajpa23819-bib-0006] Brickley, M. , Mays, S. , & Ives, R. (2010). Evaluation and interpretation of residual rickets deformities in adults. International Journal of Osteoarcheology, 20, 54–66. 10.1002/oa.1007

[ajpa23819-bib-0007] Brickley, M. , Moffat, T. , & Watamaniuk, L. (2014). Biocultural perspectives of vitamin D deficiency in the past. Journal of Anthropological Archaeology, 23, 48–59. 10.1016/j.jaa.2014.08.002

[ajpa23819-bib-0008] Chaussain‐Miller, C. , Sinding, C. , Wolikow, M. , Lasfargues, J. , Godeau, G. , & Garabédian, M. (2003). Dental abnormalities in patients with familial hypophosphatemic vitamin D‐resistent rickets: Prevention by early treatment with 1‐hydroxyvitamin D. Journal of Pediatrics, 142, 324–331. 10.1067/mpd.2003.119 12640383

[ajpa23819-bib-0009] Colombo, A. , D'Ortenzio, L. , Bertrand, B. , Coqueugniot, H. , Knüsel, C. J. , Kahlon, B. , … Brickley, M. (2019). Micro‐computed tomography of teeth as an alternative way to detect and analyse vitamin D deficiency. Journal of Archaeological Science: Reports, 23, 390–395. 10.1016/j.jasrep.2018.11.006

[ajpa23819-bib-0010] De Boer, H. H. , Aarents, M. J. , & Maat, G. J. R. (2013). Manual for the preparation and staining of embedded natural dry bone tissue sections for microscopy. International Journal of Osteoarchaeology, 23, 83–93. 10.1002/oa.1242

[ajpa23819-bib-0011] D'Ortenzio, L. , Kahlon, B. , Peacock, T. , Salahuddin, H. , & Brickley, M. (2018). The rachitic tooth: Refining the use of interglobular dentine in diagnosing vitamin D deficiency. International Journal of Paleopathology, 22, 101–108. 10.1016/j.ijpp.2018.07.001 30048808

[ajpa23819-bib-0012] D'Ortenzio, L. , Ribot, I. , Kahlon, B. , Betrand, B. , Bocaege, E. , Raguin, E. , … Brickley, M. B. (2018). The rachitic tooth: The use of radiographs as a screening technique. International Journal of Paleopathology, 23, 32–42. 10.1016/j.ijpp.2017.10.001 30573164

[ajpa23819-bib-0013] D'Ortenzio, L. , Ribot, I. , Raguin, E. , Schattmann, A. , Bertrand, B. , Kahlon, B. , … Brickley, M. (2016). The rachitic tooth: A histological examination. Journal of Archaeological Science, 74, 152–163. 10.1016/j.jas.2016.06.006

[ajpa23819-bib-0014] Dror, D. K. , & Allen, L. H. (2010). Vitamin D inadequacy in pregnancy: Biology, outomces, and interventions. Nutrition Reviews, 68, 465–477. 10.1111/j.1753-4887.2010.00306.x 20646224

[ajpa23819-bib-0015] Eckhardt, C. L. , Gernand, A. D. , Roth, D. E. , & Bodnar, L. M. (2015). Maternal vitamin D status and infant anthropometry in a US multi‐centre cohort study. Annals of Human Biology, 42, 217–224. 10.3109/03014460.2014.954616 PMC4379132 25268792

[ajpa23819-bib-0016] Eerkens, J. W. , Berget, A. G. , & Bartelink, E. J. (2011). Estimating weaning and early childhood diet from serial micro‐samples of dentin collagen. Journal of Archaeological Science, 38, 3101–3111. 10.1016/j.jas.2011.07.010

[ajpa23819-bib-0017] Foster, B. L. , Nociti, F. H. , & Somerman, M. J. (2014). The rachitic tooth. Endocrine Reviews, 35, 1–34. 10.1210/er.2013-1009 23939820 PMC3895863

[ajpa23819-bib-0018] Giuffra, V. , Vitiello, A. , Caramella, D. , Fornaciari, A. , Giustini, D. , & Fornaciari, G. (2015). Rickets in a high social class of renaissance Italy: The Medici children. International Journal of Osteoarchaeology, 25, 608–624. 10.1002/oa.2324

[ajpa23819-bib-0019] Gustafson, G. , & Koch, G. (1974). Age estimation up to 16 years of age based on dental development. Odontology Reviews, 25, 297–306. 4530955

[ajpa23819-bib-0020] Hess, A. F. (1930). Rickets. Including osteomalacia and tetany. London: Kimpton.

[ajpa23819-bib-0021] Hillson, S. (2002). Dental anthropology. Cambridge, UK: Cambridge University Press.

[ajpa23819-bib-0022] Holick, M. F. (2003). Vitamin D: A millennium perspective. Journal of Cellular Biochemistry, 88, 296–307. 10.1002/jcb.10338 12520530

[ajpa23819-bib-0023] Holick, M. F. (2004). Vitamin D: Importance in the prevention of cancers, type 1 diabetes, heart disease, and osteoporosis. American Journal of Clinical Nutrition, 79(3), 362–371. 10.1093/ajcn/79.3.362 14985208

[ajpa23819-bib-0024] Holick, M. F. (2006). Resurrection of vitamin D deficiency and rickets. Journal of Clinical Investigation, 116, 2062–2072. 10.1172/JCI29449 16886050 PMC1523417

[ajpa23819-bib-0025] Isokawa, S. , Kosakai, T. , & Kajiyama, S. (1963). Interglobular dentine in the deciduous tooth. Journal of Dental Research, 42, 831–834. 10.1177/00220345630420031301 13956926

[ajpa23819-bib-0026] Jablonski, N. G. , & Chaplin, G. (2013). Epidermal pigmentation in the human lineage is an adaptation to ultraviolet radiation. Journal of Human Evolution, 65, 671–675. 10.1016/j.jhevol.2013.06.004 24112698

[ajpa23819-bib-0027] Jablonski, N. G. , & Chaplin, G. (2018). The roles of vitamin D and cutaneous vitamin D production in human evolution and health. International Journal of Paleopathology, 23, 54–59. 29606375 10.1016/j.ijpp.2018.01.005

[ajpa23819-bib-0029] Lovejoy, C. O. (1985). Dental wear in the Libben population: Its functional pattern and role in the determination of adult skeletal age at death. American Journal of Physical Anthropology, 68, 47–56. 10.1002/ajpa.1330680105 4061601

[ajpa23819-bib-0030] Massler, M. , Schour, L. , & Poncher, H. G. (1941). Developmental pattern of the child as reflected in the calcification pattern of the teeth. American Journal of Diseases of Children, 62, 33–67. 10.1001/archpedi.1941.02000130042004

[ajpa23819-bib-0031] Mays, S. , Brickley, M. , & Ives, R. (2006). Skeletal manifestations of rickets in infants and young children in a historic population from England. American Journal of Physical Anthropology, 129, 362–374. 10.1002/ajpa.20292 16323190

[ajpa23819-bib-0032] Mays, S. , Brickley, M. , & Ives, R. (2009). Growth and vitamin D deficiency in a population from 19th century Birmingham, England. International Journal of Osteoarchaeology, 19, 406–415. 10.1002/oa.976

[ajpa23819-bib-0033] McDonnell, D. , Derkson, G. , Zhang, L. , & Hlady, J. (1997). Nutirional rickets in a 2‐year‐old child: Case report. Pediatric Dentistry, 19, 127–130. 9106876

[ajpa23819-bib-0034] Moorrees, C. , Fanning, E. , & Hunt, E. (1963). Age variation of formation stage for ten permanent teeth. Journal of Dental Research, 42, 1490–1502. 10.1177/00220345630420062701 14081973

[ajpa23819-bib-0035] Mulligan, M. L. , Felton, S. K. , Riek, A. E. , & Bernal‐Mizrachi, C. (2010). Implications of vitamin D deficiency in pregnancy and lactation. American Journal of Obstetrics & Gynecology, 429, e1–e9. 10.1016/j.ajog.2009.09.002 PMC3540805 19846050

[ajpa23819-bib-0036] Ortner, D. J. , & Mays, S. (1998). Dry‐bone manifestations of rickets in infancy and early childhood. International Journal of Osteoarchaeology, 8, 45–55. 10.1002/(SICI)1099-1212(199801/02)8:1<45::AID-OA405>3.0.CO;2-D

[ajpa23819-bib-0037] Palkovich, A. M. (2012). Reading a life: A fourteenth‐century ancestral Puebloan woman In Stodder A. L. W. & Palkovich A. M. (Eds.), Bioarchaeology of individuals (pp. 242–254). Florida: University Press of Florida.

[ajpa23819-bib-0038] Ribeiro, T. R. , Costa, F. W. G. , Soares, E. C. S. , Williams, J. R., Jr. , & Fonteles, C. S. R. (2015). Enamel and dentin mineralization in familial hypophosphatemic rickets: A micro‐CT study. Dentomaxillofacial Radiology, 44, 20140347 10.1259/dmfr.20140347 25651274 PMC4628496

[ajpa23819-bib-0040] Souza, M. A. , Soares, L. A. V., Jr. , Alves dos Santos, M. , & Vaisbisch, M. H. (2010). Dental abnormalities and oral health in patients with hypophosphatemic rickets. Clinics, 65, 1023–1026. 10.1590/S1807-59322919991999917 21120305 PMC2972601

[ajpa23819-bib-0041] Thandrayen, K. , & Pettifor, J. M. (2010). Maternal vitamin D status: Implications for the development of infantile nutritional rickets. Endocrinology and Metabolism Clinics of North America, 39, 303–320. 10.1016/j.ecl.2010.02.006 20511053

[ajpa23819-bib-0042] Veselka, B. (2019). *D‐lightful sunshine disrupted: Vitamin D deficiency as a method for the reconstruction of changes in sociocultural practices due to industrialisation in 17th–19th century Netherlands* (Published doctoral thesis). Leiden University. Leiden. Retreived from https://openaccess.leidenuniv.nl/handle/1887/68401.

[ajpa23819-bib-0043] Veselka, B. , Hoogland, M. L. P. , & Waters‐Rist, A. L. (2015). Rural rickets: Vitamin D deficiency in a post‐medieval farming community from The Netherlands. International Journal of Osteoarchaeology, 25, 665–675. 10.1002/oa.2329

[ajpa23819-bib-0044] Veselka, B. , Hoogland, M. L. P. , & Waters‐Rist, A. L. (2018). Gender‐related vitamin D deficiency in a Dutch 19th century farming community. International Journal of Paleopathology, 23, 69–75. 10.1016/j.ijpp.2017.11.001 30573168

[ajpa23819-bib-0045] Vital, S. O. , Gaucher, C. , Bardet, C. , Rowe, P. S. , George, A. , Linglart, A. , … Chaussain, C. (2012). Tooth dentin defects reflect genetic disorders affecting bone mineralization. Bone, 50, 989–997. 10.1016/j.bone.2012.01.010 22296718 PMC3345892

[ajpa23819-bib-0046] Waters‐Rist, A. L. , & Hoogland, M. L. P. (2018). The role of infant feeding and childhood diet in vitamin D deficiency in a nineteenth century rural Dutch community. Bioarchaeology International, 2, 95–116.

[ajpa23819-bib-0047] Watts, R. , & Valme, S. R. (2018). Osteological evidence for juvenile vitamin D deficiency in a 19th century suburban population from Surrey, England. International Journal of Paleopathology, 23, 60–68. 10.1016/j.ijpp.2018.01.007 30573167

[ajpa23819-bib-0048] Webb, A. R. , Kline, L. , & Holick, M. F. (1988). Influence of season and latitude on the cutaneous synthesis of vitamin D3: Exposure to winter sunlight in Boston and Edmonton will not promote vitamin D3 synthesis in human skin. Journal of Clinical Endocrinology and Metabolism, 67, 373–378. 10.1210/jcem-67-2-373 2839537

