# Supplementary material for: Micro‐CT assessment of dental mineralization defects indicative of vitamin D deficiency in two 17th–19th century Dutch communities
Source: Am J Phys Anthropol. 2019 Mar 18;169(1):122–31. doi: 10.1002/ajpa.23819 (PMC6593783; doi:10.1002/ajpa.23819)
Supplement: Supplementary file 1 — Appendix S1: Supporting information [file AJPA-169-122-s001.docx]

| Individual |  |  |  |  | |  | Bending deformities | | | | | | | | |
| --- | --- | --- | --- | --- | --- | --- | --- | --- | --- | --- | --- | --- | --- | --- | --- |
|  | Age  (years) | Sex | DCP | | ORP | EH | | Femur | Tibia | Fibula | Humerus | Radius | Ulna | Clavicle | Ribs |
| HT15S020 | 15 ± 2 | U |  | |  |  | | X |  |  |  |  |  |  |  |
| HT15S042 | 36 – 49 | M | X | |  |  | | X (L) | X |  |  |  |  |  |  |
| HT15S062 | 36 – 49 | F |  | |  |  | | X (L) | X |  |  |  |  |  |  |
| HT15S066 | 36 – 49 | M |  | | X |  | |  | X | X (L) |  |  |  |  |  |
| HT15S067 | 6.5 ± 0.5 | U |  | |  |  | |  | X | X |  |  |  |  |  |
| HT15S071 | 18 – 25 | M |  | |  |  | | X | X |  |  |  |  |  |  |
| HT15S075 | 36 – 49 | F |  | |  | X | | X |  |  | X |  |  | X | X |
| HT15S080 | 26 – 35 | M | X | |  |  | | X |  | X |  |  |  |  |  |
| HT15S094 | 18 – 25 | F |  | |  |  | | X | X |  | X | X | X |  |  |
| HT15S099 | 18 – 25 | F |  | | X |  | | X | X |  |  |  |  |  |  |
| HT15S106 | 36 – 49 | M |  | | X | X | |  | X |  |  |  |  |  |  |
| HT15S109 | 36 – 49 | F |  | |  |  | | X | X |  |  |  |  |  |  |
| HT15S123 | 2 ± 0.5 | U |  | | X |  | | X | X | X | X | X | X |  | X |
| HT15S127 | 9 ± 1 | U |  | |  | X | | X | X |  |  | X |  |  |  |
| HT15S130 | 18 – 25 | M | X | |  | X | | X | X | X |  | X | X |  |  |
| MB11S101 | 26 – 35 | F |  | |  |  | | X | X |  |  |  |  |  |  |
| MB11S126 | 36 – 49 | F |  | |  |  | |  |  |  |  | X | X |  |  |
| MB11S183 | 26 – 35 | F |  | |  |  | |  |  | X | X |  |  |  |  |
| MB11S234 | 18 – 25 | F |  | |  |  | | X |  |  |  |  |  |  |  |
| MB11S307 | 18 – 25 | F |  | |  |  | | X | X | X |  |  |  |  |  |
| MB11S321 | 50+ | M |  | |  |  | |  | X |  |  |  |  |  |  |
| MB11S327 | 26 – 35 | F |  | |  |  | | X | X |  | X |  |  |  |  |
| MB11S401 | 26 – 35 | F |  | |  |  | | X | X | X |  |  |  |  |  |
| MB11S413 | 36 – 49 | F |  | |  |  | | X (L) |  |  |  | X (L) |  |  |  |
| MB11S420 | 26 – 35 | F |  | |  |  | | X | X |  |  |  |  |  |  |
| MB11S422 | 36 – 49 | F |  | |  |  | |  | X |  |  | X |  |  |  |
| MB11S427 | 26 – 35 | M |  | |  |  | | X | X | X |  |  |  |  |  |
| MB11S437 | 26 – 35 | F |  | |  |  | | X |  |  |  |  |  |  |  |
| MB11S488 | 36 – 49 | F |  | |  |  | | X | X | X |  |  |  |  |  |
| MB11S498 | 50+ | F |  | |  |  | | X | X |  |  |  |  |  |  |

**Appendix**. Overview of macroscopic lesions per individual that may be attributed to vitamin D deficiency. HT = Hattem, MB = Beemster, DCP = diffuse cranial vault porosity, ORP = orbital roof porosity, EH = enamel hypoplasia, X = present bilateral unless indicated otherwise, L = left.
